# New Type of Sendai Virus Vector Provides Transgene-Free iPS Cells Derived from Chimpanzee Blood

**DOI:** 10.1371/journal.pone.0113052

**Published:** 2014-12-05

**Authors:** Yasumitsu Fujie, Noemi Fusaki, Tomohiko Katayama, Makoto Hamasaki, Yumi Soejima, Minami Soga, Hiroshi Ban, Mamoru Hasegawa, Satoshi Yamashita, Shigemi Kimura, Saori Suzuki, Tetsuro Matsuzawa, Hirofumi Akari, Takumi Era

**Affiliations:** 1 Department of Cell Modulation, Institute of Molecular Embryology and Genetics, Kumamoto University, 2-2-1 Honjo, Chuo-ku, Kumamoto 860-0811, Japan; 2 DNAVEC Corporation, 6 Ookubo, Tsukuba, Ibaragi 300-2611, Japan; 3 Precursory Research for Embryonic Science and Technology, Japan Science and Technology Agency, 4-1-8 Honcho Kawaguchi, Saitama, Japan; 4 Department of Neurology, Graduate School of Medical Sciences, Kumamoto University, 1-1-1 Honjo, Chuo-ku, Kumamoto 860-8556, Japan; 5 Department of Child Development, Graduate School of Medical Sciences, Kumamoto University, 1-1-1 Honjo, Chuo-ku, Kumamoto 860-8556, Japan; 6 Section of Comparative Microbiology and Immunology, Center for Human Evolution Modeling Research, Primate Research Institute, Kyoto University, Inuyama, Aichi 484-8506, Japan; 7 Section of Language and Intelligence, Primate Research Institute, Kyoto University, Inuyama, Aichi 484-8506, Japan; 8 Laboratory of Evolutional Virology, Experimental Research Center for Infectious Diseases, Institute for Virus Research, Kyoto University, Kyoto, 606-8507, Japan; Rutgers University -New Jersey Medical School, United States of America

## Abstract

Induced pluripotent stem cells (iPSCs) are potentially valuable cell sources for disease models and future therapeutic applications; however, inefficient generation and the presence of integrated transgenes remain as problems limiting their current use. Here, we developed a new Sendai virus vector, TS12KOS, which has improved efficiency, does not integrate into the cellular DNA, and can be easily eliminated. TS12KOS carries *KLF4, OCT3/4*, and *SOX2* in a single vector and can easily generate iPSCs from human blood cells. Using TS12KOS, we established iPSC lines from chimpanzee blood, and used DNA array analysis to show that the global gene-expression pattern of chimpanzee iPSCs is similar to those of human embryonic stem cell and iPSC lines. These results demonstrated that our new vector is useful for generating iPSCs from the blood cells of both human and chimpanzee. In addition, the chimpanzee iPSCs are expected to facilitate unique studies into human physiology and disease.

## Introduction

Induced pluripotent stem cells (iPSCs) artificially produced from mammalian somatic cells including mouse and rat, human, marmoset, rhesus monkey, and pig can be induced to undergo sustained, unlimited growth and give rise to various cell types *in vitro* [1]–[7]. Because of these features, iPSCs have important potential applications as a source for cell therapy in clinical medicine. The iPSCs derived from patients also represent a powerful tool both for understanding disease pathogenesis and for investigating the effects of drugs at the individual and disease level on patient-derived cells [8].

While iPSCs derived from a range of mammalian species could serve as useful translational and disease models for cell and drug therapies, cell lines derived from nonhuman primates are particularly useful for such studies because the anatomical and physiological features of these species tend to be more similar to human than those of other mammals [9], [10]. Among nonhuman primates, the chimpanzee is often not a suitable experimental model because of breeding limitations; however, the chimpanzee shares some important physiological features with humans such as surface antigen cross-recognition by antibodies [11], [12]. Chimpanzees and humans occasionally share common pathogens, including ebola virus and hepatitis virus type B [13]. Thus, chimpanzee cells and stem cells derived from them represent powerful tools for both research and clinical applications in human disease.

The process of iPS cell generation, known as reprogramming, is triggered by the expression of four transcription factors, Oct3/4, Sox2, Klf4, and c-Myc, which are the same core factors underlying pluripotency in other stem cells such as embryonic stem cells (ESCs) [14]. Since overexpression of the four factors was initially mediated by lentivirus and retrovirus vectors in human skin-derived fibroblasts [5], [15], many methods have been reported including those involving transposons [16], proteins [17], microRNAs [18], and plasmids [19]. Recent progress has seen the increasing use of either plasmids or Sendai virus (SeV) vectors to generate iPS cells easily and quickly from human peripheral blood cells [20], [21]. Both methods are simple to conduct compared to other procedures, and are safer because there is no integration of transgenes into the host genome; however, the frequency of iPS cell colony generation remains low (∼0.1%) and it is difficult to attain completely vector-free iPSCs.

Here, we generated a new SeV vector that enables highly efficient generation of iPS cells from peripheral blood cells, is temperature-sensitive (TS), and is quickly and efficiently eliminated from the established iPSCs by temperature shift. In addition to the generation of human iPSCs, we succeeded in establishing iPSC lines derived from the blood cells of chimpanzees.

## Results

### Vector generation

**Figure 1:**
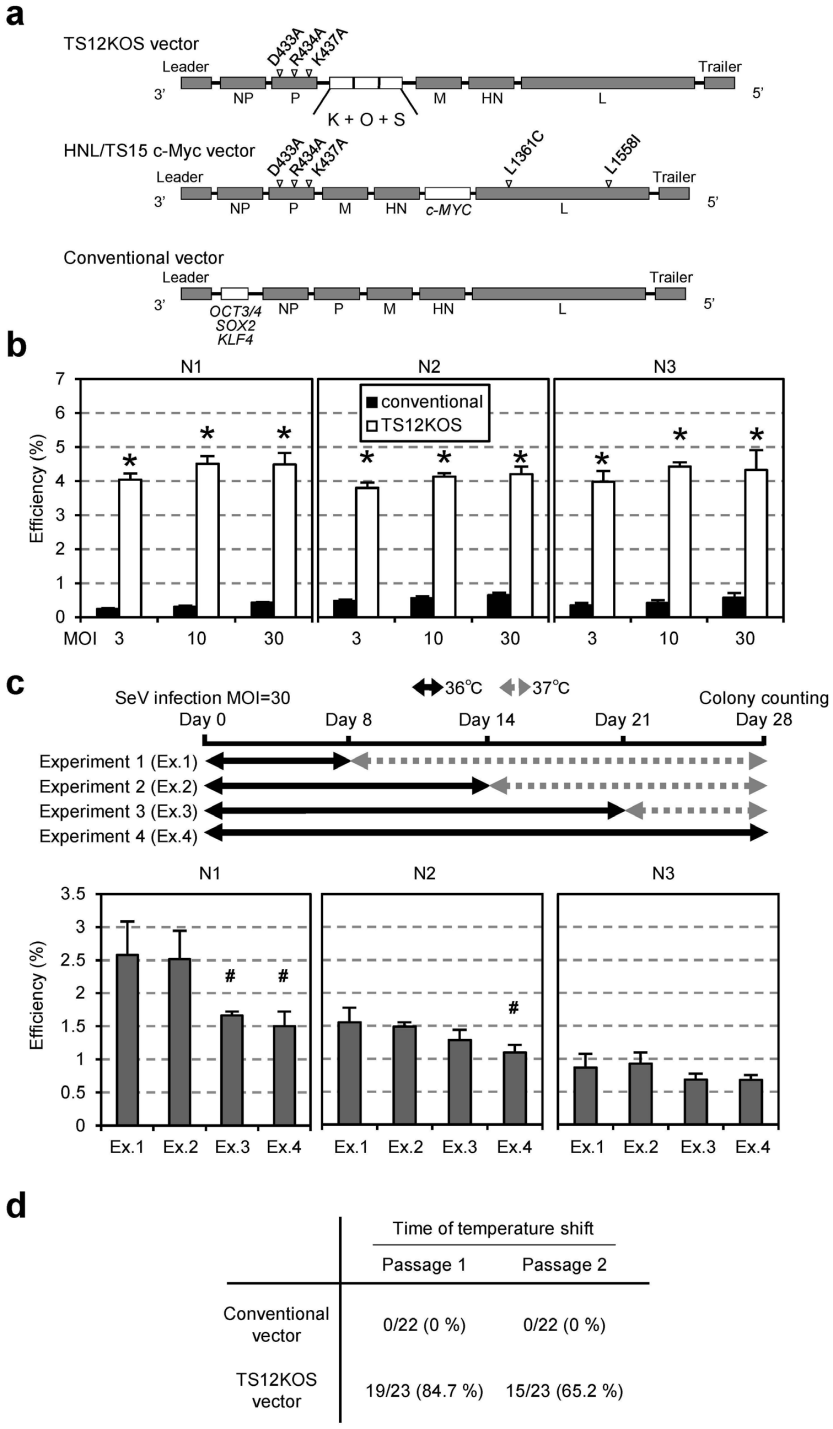
Generation of a new temperature-sensitive Sendai virus vector, TS12KOS. (**a**) Comparison of schematic structures among the newly constructed Sendai virus (SeV) vector, TS12KOS, and previous vectors. The TS12KOS vector contains three point mutations in the RNA polymerase–related gene (P) and carries the coding sequences of *KLF4* (K), *OCT3/4* (O), and *SOX2* (S) in the KOS direction. In comparison, the HNL/TS15 c-Myc vector carries two additional mutations, L1361C and L1558I, in the large polymerase (L) gene and an exogenous c-*MYC* cDNA sequence inserted between the hemagglutinin-neuraminidase (HN) and L genes, and the conventional vectors individually carry three reprogramming factors as indicated. (**b**) iPS cell generation from human skin-derived fibroblasts. The efficiency of iPS cell generation was significantly higher using the TS12KOS vector than with the conventional vectors at all multiplicities of infection (MOI) tested. iPSC colonies were identified on day 28 of induction by the appearance of alkaline phosphatase-positive (AP^+^) colonies with embryonic stem (ES) cell-like colony morphology. N1, N2, and N3 represent individual healthy volunteers. Experiments were conducted in triplicate (mean ± SD). *P<0.01, TS12KOS vector versus conventional vectors, Student's t-test. (**c**) Temperature shift from 37°C to 36°C for the indicated periods in iPSC generation. Data are means ± SD of three independent experiments. ^#^ *P*<0.05, Experiment 2, 3 and 4 versus Experiment 1. Student's t-test. (**d**) Nested RT-PCR analysis of SeV vector elimination after the temperature shift from 37°C to 38°C in human fibroblast-derived iPSCs. The elimination of TS12KOS vector was faster than the conventional vectors.

We previously generated iPSCs by using SeV vectors containing the sequences for four reprogramming factors (*OCT3/4*, *SOX2*, *KLF4* and *c-MYC*) [22]. Here, to increase the efficiency of iPSC generation and reduce the length of time the vector remains inside the cells, we generated a new TS-SeV vector, TS12KOS, carrying coding sequences for three of the above factors, *KLF4* (K), *OCT3/4* (O), and *SOX2* (S) (**Fig. 1a**) tandemly linked in the KOS direction. The TS12KOS vector contains three mutations that produce alanine residues (D433A, R434A, and K437A) in the large protein (L)-binding domain of the phosphoprotein (P), a component of SeV RNA polymerase. SeV carrying these three mutations showed moderate expression of GFP at 37°C, but weak expression at temperatures above 38°C [23]. In a previous study, c-*MYC* was inserted between the sequences encoding the HN and L proteins in the TS15 SeV vector (HNL/TS15 c-MYC), which carries two other mutations (L1361C and L1558I) in addition to the triple mutation described above [23]. This vector is also temperature-sensitive and only weakly expressed at temperatures greater than 37°C. In this study, TS12KOS vector and a cocktail of conventional vectors carrying three reprogramming factors individually (*OCT3/4*, *SOX2* and *KLF4*), namely the conventional vectors, were used with HNL/TS15 c-MYC in following experiments.

First, we compared TS12KOS with the conventional SeV vectors for the efficiency of iPSC generation from human skin fibroblasts of healthy volunteers (**Fig. 1b**). Based on the numbers of colonies showing alkaline phosphatase (AP)-positive staining and human ESC-like morphology on day 28 after induction, we found that the efficiency of iPSC generation was significantly higher using the TS12KOS vector than with the conventional vectors.

We next examined the effect of temperature shift on iPSC generation from human fibroblasts. When the culture temperature was shifted from 37°C to 36°C for the initial two weeks after infection, the efficiency of colony formation remained high; however, when the temperature downshift continued for three weeks or more after infection, the efficiency tended to decrease in the samples from healthy volunteers (**Fig. 1c**). Therefore, a temperature downshift for the initial one week only was used in the following experiments.

We next conducted nested RT-PCR analysis of viral RNA to determine whether the TS12KOS vector was eliminated from the iPSCs earlier than the conventional SeV vectors. The nested RT-PCR analysis detects the viral genome with much higher sensitivity than single PCR [24]. We expanded the individual colonies and shifted the temperature from 37°C to 38°C for 3 days at various passages. In the conventional SeV infections, temperature upshifts at passage 1 or 2 induced no virus removal. In contrast, when the temperature of TS12KOS vector-infected cells was upshifted at passages 1 and 2, 84% and 65%, respectively, of iPSC-like clones were negative for viral genomic nucleic acid (**Fig. 1d**). These results indicated the TS12KOS vector superiority over the conventional SeV vectors in both efficiency of iPSC generation and elimination of virus from the iPSCs.

Based on previous findings that L-*MYC* is safer than c-*MYC* due to a lower incidence of tumorigenicity, we next examined the effect of replacing the c-*MYC* cDNA sequences with L-*MYC* cDNA sequences in the HNL/TS15 c-MYC SeV vector (**Fig. S1a**) [25]. The frequency of colonies with ALP+ and ESC-like morphology was lower using the L-*MYC* vector than with the original HNL/TS15 c-MYC vector (**Fig. S1b**), despite the L-*MYC* gene showing higher expression levels (data not shown).

Because Glis1 can enhance iPSC generation, we also constructed and tested various SeV vectors carrying *GLIS1* sequences (**Fig. S1a, c**) [26]. Unexpectedly, Glis1 expression did not augment the colony formation from human skin-derived fibroblasts with or without c-Myc, suggesting that Glis1 does not play a part in iPSC induction with SeV vector (**Fig. S1c**).

### Characterization of human iPS cells generated with new virus vector

**Figure 2:**
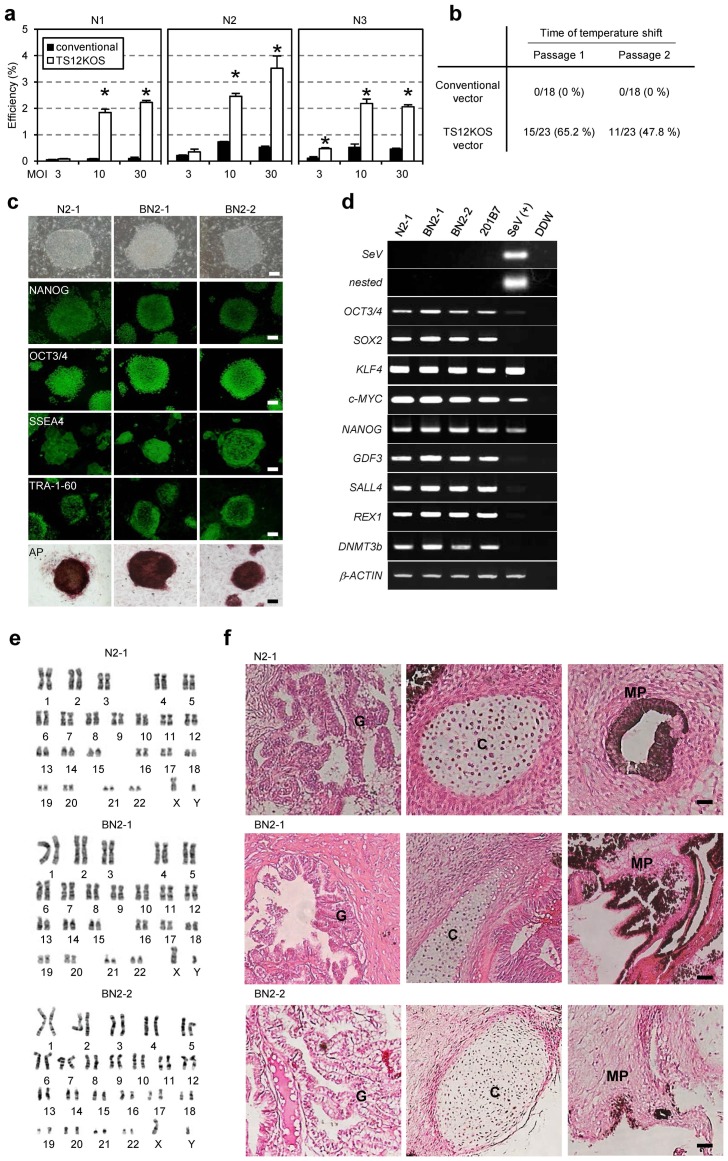
Characterization of human iPSCs generated by the TS12KOS vector. (**a**) iPSC generation from human peripheral blood cells. Experiments were conducted in triplicate (mean ± SD). N1, N2, and N3 indicate individual healthy volunteers. *P<0.01, TS12KOS vector versus conventional vectors, Student's t-test. (**b**) Nested RT-PCR analysis of the elimination of SeV vectors after the temperature shift from 37°C to 38°C. (**c**) Phase contrast images, immunofluorescence for pluripotency markers, and alkaline phosphatase (AP) staining of iPSC lines. The iPSC lines N2-1 and BN2-1 and BN2-2 were derived from the skin-derived fibroblasts and blood cells of N2 healthy volunteer, respectively. Scale bars, 200 µm. (**d**) RT-PCR analysis of Sendai virus and human ES cell markers. SeV, first RT-PCR for SeV; nested, nested RT-PCR for SeV; 201B7, control human iPSC line; SeV(+), Day 7 SeV-infected human fibroblasts. (**e**) Chromosomal analyses of iPSC lines generated with the TS12KOS vector. (**f**) Tissue morphology of a representative teratoma derived from iPSC lines generated with the TS12KOS vector. G, glandular structure (endoderm); C, cartilage (mesoderm); MP, melanin pigment (ectoderm). Scale bars, 100 µm.

Our ultimate goal is to develop safe and efficient vectors to generate iPSCs from both human and primate peripheral blood cells. When we stimulated human peripheral T lymphocytes with both anti-CD3 antibody and interleukin 2, and then infected them with SeV vectors, iPSC generation was significantly more efficient using the TS12KOS vector than with the conventional SeV vectors (**Fig. 2a**). In conventional SeV infections, temperature shifts from 37°C to 38°C at passages 1 and 2 induced no elimination of virus from the iPSC clones (**Fig. 2b**). In contrast, when TS12KOS vector was used under the same conditions, 65% and 47%, respectively, of the clones were negative for viral genome (**Fig. 2b**). Therefore, similar to the results obtained with fibroblasts, the elimination of TS12KOS vector from iPS-like cells derived from peripheral T lymphocytes was faster than that observed for the conventional SeV vectors.

The iPSC lines derived from skin fibroblasts and peripheral T lymphocytes induced by TS12KOS vector exhibited a typically ESC-like morphology and expressed a set of typical markers for pluripotency (**Fig. 2c, d**). These iPSC lines had a normal 46 XY karyotype even after the temperature upshift and culturing for more than 10 passages (**Fig. 2e**). To confirm the pluripotency of the clonal lines, we transplanted the lines into the testis of immunodeficient mice. Twelve weeks after injection, the iPSC lines tested formed teratomas that contained derivatives of all three germ layers (**Fig. 2f**). Based on these findings, we conclude that the iPSC lines generated with TS12KOS vector meet the criteria of iPSCs.

### Establishment of chimpanzee iPS cells

**Figure 3:**
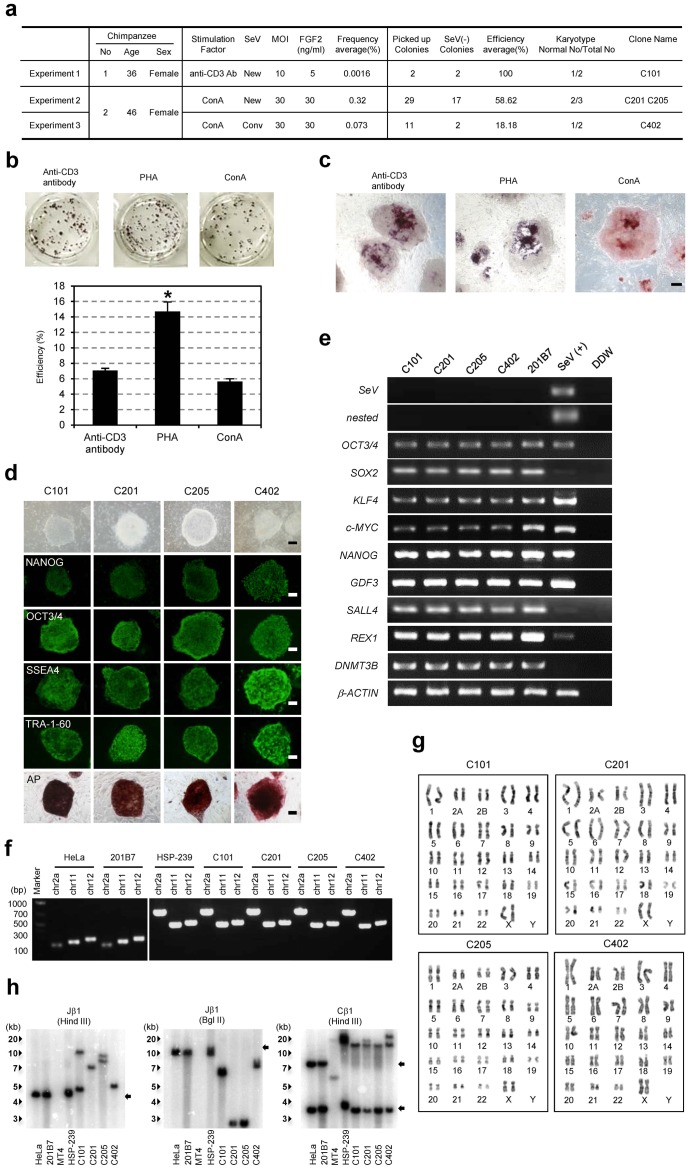
Generation of chimpanzee iPSCs with the TS12KOS vector. (**a**) Summary of chimpanzee iPSC generation. iPSCs were generated from the blood cells of two chimpanzee individuals with TS12KOS or the conventional SeV vectors. (**b**) Effect of the T lymphocyte stimulation on iPSC generation. Experiments were conducted in triplicate (mean ± SD). *P<0.01, PHA versus anti-CD3 antibody or Con A stimulations, Student's t-test. (**c**) Colony morphology and AP staining of iPSCs from stimulated T lymphocytes. (**d**) Phase contrast images, immunofluorescence for pluripotency markers, and alkaline phosphatase (AP) staining of chimpanzee iPSC lines. C101, C201, C205, and C402 are described in **Fig. 3a**. Scale bars, 200 µm. (**e**) RT-PCR analysis of SeV and human ES cell markers. SeV, first RT-PCR for SeV; nested, nested RT-PCR for SeV; 201B7, control human iPSC line; SeV(+), Day 7 SeV-infected human fibroblasts. (**f**) PCR products with primers that can distinguish chimpanzee and human genomes. Chimpanzee PCR products; 782, 472 and 504 bps, Human PCR products; 203, 245, 278 bps. (**g**) Chromosomal analyses of chimpanzee iPSC lines generated with the TS12KOS vector. (**h**) TCR gene recombination. Genes from the chimpanzee iPSC lines were digested with the indicated enzymes and hybridized with the TCR probes by Southern blotting. Arrows indicate the germ bands of TCR genes. HeLa and 201B7: human cell lines, MT4: human T cell line, HSP-239: chimpanzee T cell line.

Next we used the TS12KOS vector to establish iPSC lines from the blood cells of two chimpanzee individuals, with the ultimate goal of overcoming the limited availability of chimpanzee skin-fibroblasts for human medical use. Using the same protocol as for human blood cells, we could establish chimpanzee blood cell-derived iPSCs (**Fig. 3a**). However, the frequency was relatively low and only one cell line that carries the normal karyotype could be established (Experiment 1 in **Fig. 3a**). To optimize the induction conditions, we conducted *in vitro* human T lymphocyte stimulations with anti-CD3, Phytohaemagglutinin (PHA), or Concanavalin A (Con A), and similarly generated iPSCs from human peripheral mononuclear cells (PMNCs) using all three agents, with PHA stimulation the most efficient (**Fig. 3b**). The morphology of iPSC colonies derived from the anti-CD3- and PHA-stimulated PMNCs was distinct from colonies derived from the Con A-stimulated PMNCs (**Fig 3c**), which produced flat colonies with clearer and sharper edges than those derived from CD3- and PHA-stimulated PMNCs. In addition, many of the iPSC colonies derived from CD3- and PHA-stimulated PMNCs contained AP^+^ cells in the center only (**Fig. 3c**). Together, these results suggested that colonies derived from Con A-stimulated PMNCs most closely fulfill the accepted criteria of iPSCs. Therefore, Con A was used in the following experiment 2 and 3 (**Fig. 3a**).

In addition, we changed the virus titer for infection from MOI 10 to 30 and the fibroblast growth factor 2 (FGF2) concentration from 5 ng/ml to 30 ng/ml during the iPSC induction (**Fig. S2**). The FGF2 change was based on a study of common marmosets, another nonhuman primate, which used 20 ng/ml FGF2 and treated the cultures with sodium butyrate (NaB) during reprogramming to enhance the iPSC colony number [7], [27]. In human blood cells, the efficiency of iPSC generation with 30 ng/ml FGF2 is slightly but not significantly lower than that with 5 ng/ml FGF2 (**Fig. S3**). Interestingly, these modifications improved the colony frequency and provided many more iPSC colonies during the second round of iPSC induction (Experiment 2 in **Fig. 3a**). However, the efficiency of iPSC generation from chimpanzee PMNCs was still lower than that from human blood cells (0.32% vs. 2%, **Fig. 3a**). We tried picking up the colonies again and expanded them, before shifting the culturing temperature to 38°C for three days at passage 1 to eliminate the Sendai virus (**Fig. 3a**). The rate of virus elimination from the chimpanzee iPSCs was similar to that from human iPSCs (65.2% vs. 58.6%).

We then compared the efficiency of iPSC generation with TS12KOS vector to that with the conventional SeV vectors (Experiment 3 in **Fig. 3a**). Although a high titer (MOI 30) of the conventional SeV vectors could generate chimpanzee iPSCs, TS12KOS vector showed a higher efficiency of iPSC generation (0.073% for the conventional vectors vs. 0.32%, **Fig. 3a**). Similarly, the elimination rate of SeV was lower than that observed with the conventional vectors, suggesting that our new vector could more efficiently generate the transgene-free iPSCs from chimpanzee blood cells than the conventional SeV vectors.

The chimpanzee iPSC lines exhibited ESC-like morphology and expressed a set of pluripotent markers (**Fig. 3d, e**), with nested RT-PCR analysis determining that the iPSC lines were negative for SeV genomic material (**Fig. 3e**). To confirm that these cells were truly derived from chimpanzee, we also performed genomic PCR using chimpanzee-specific primers (**Fig. 3f**), with different PCR product sizes allowing us to easily distinguish between chimpanzee and human genes and confirming that the chimpanzee-derived samples contained no human DNA fragments [28]. Karyotype analyses showed that the iPSC lines had a normal karyotype, 48XX, further confirming that the iPSC lines were derived from chimpanzee (**Fig. 3g**).

To investigate the cellular origin of the chimpanzee iPSCs, we performed Southern blot analyses with probes specific for T cell receptor (TCR) DNA (**Fig. 3h**) [29]. Rearranged bands were detected in all iPSC lines, indicating that they were derived from the chimpanzee T lymphocytes.

### Characterization of chimpanzee iPSC lines

**Figure 4:**
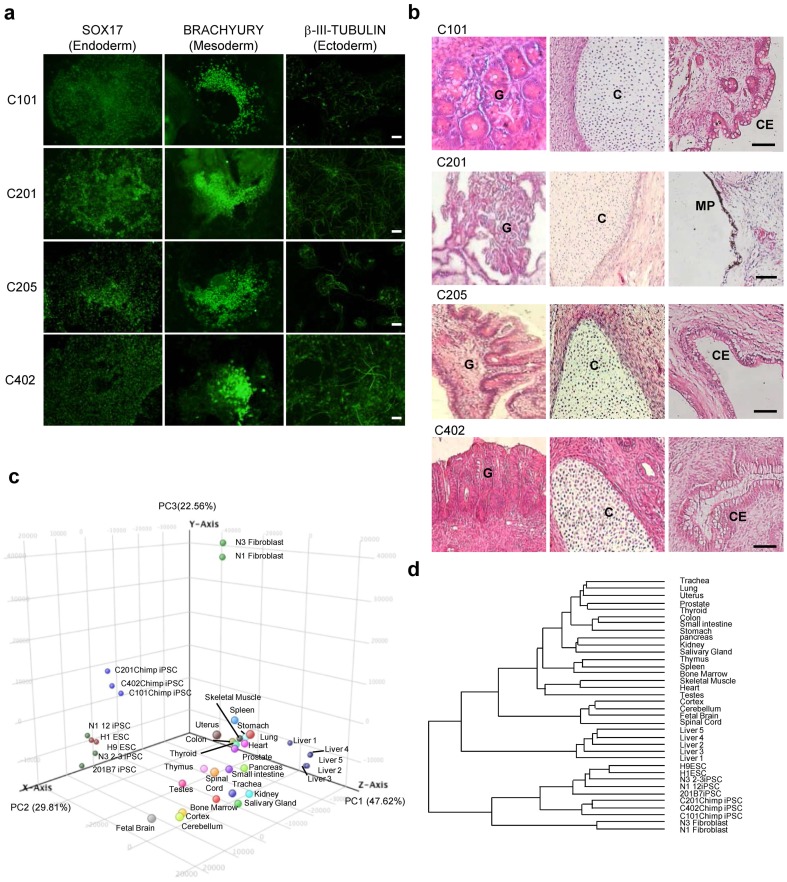
Characterization of chimpanzee iPSCs. (**a**) Differentiation into three germ layers in vitro. The chimpanzee iPSC lines can generate SOX17^+^ (endoderm), BRACHYURY^+^ (mesoderm), and βIII-tubulin^+^ (ectoderm) cells. Scale bars, 100 µm. (**b**) Tissue morphology of a representative teratoma derived from the chimpanzee iPSC lines generated with TS12KOS vector. G, glandular structure (endoderm); C, cartilage (mesoderm); CE, Cuboidal Epithelium structure (ectoderm); MP, melanin pigment (ectoderm). Scale bars, 100 µm. (**c**) Principal Component Analysis. All data sets were classified into three principal components, PC1 (47.62%), PC2 (29.81%), and PC3 (22.56%), and then simplified into three-dimensional scores. Percentage shows the portion of variance in each component. The position of chimpanzee iPSC lines is closely placed to that of human ESCs and iPSCs. (**d**) Hierarchical clustering of chimpanzee iPSCs, human iPSCs and ESCs. The data sets of all genes investigated were clustered according to Euclidean distance metrics. The data sets of chimpanzee iPSCs, human ESC and iPSC lines, and various human tissues were classified into separate branches. The datasets of human ESCs and various tissues referred for Gene Expression Omnibus datasets, GSE22167 and GSE33846, respectively.

Finally, we investigated the differentiation potential of the chimpanzee iPSCs lines by evaluating in vitro differentiation and teratoma formation (**Fig. 4a, b**). The appropriate culture conditions induced differentiation into cells representing all three germ layers, ectoderm (βIII-tubulin-positive), mesoderm (BRACHYURY-positive), and endoderm (SOX17-positive) (**Fig. 4a**). Consistent with this finding, histological analysis revealed that formed teratomas contained descendant markers of all three germ cell layers such as cuboidal epithelia, melanin pigment, cartilage, muscle, and various glandular structures (**Fig. 4b**). Taken together, the chimpanzee-derived iPSC lines fulfilled the criteria for iPSCs.

We used microarray analysis to further characterize the chimpanzee iPSC lines. The patterns of global gene expression of three chimpanzee iPSC lines were similar to those of human iPSC lines (**Fig. 4c**). Principal component analysis (PCA) and hierarchical clustering of all genes was conducted to determine overall differences in transcription levels between chimpanzee- and human-derived iPSC lines. Data from human ESCs, iPSCs, fibroblasts, and various tissues were analyzed together with those from chimpanzee iPSCs. Although the chimpanzee iPSC lines were derived from different individuals, their data were grouped by PCA and placed close to those of human ESCs and iPSCs (**Fig. 4c**). The gene-expression profiles of chimpanzee iPSCs were grouped closely to human ESCs and iPSCs in the same branch, and distinctly separated from the branch containing gene profiles of various human tissues (**Fig. 4d**). These results suggested that the global gene expression patterns of chimpanzee iPSC lines are generally similar to those of human ESCs and iPSCs.

## Discussion

We developed a new temperature-sensitive SeV vector, TS12KOS, and herein demonstrated it to be an efficient tool for generating iPSCs from both human skin fibroblasts and peripheral blood cells. Using this vector, we also generated chimpanzee iPSC lines from peripheral blood cells.

The iPSCs established with our TS12KOS vector could be made virus-free simply by shifting the temperature from 37°C to 38°C for 3 days, and transgene-free iPSCs could be generated within a week of isolating the iPSC colonies. Unlike previous techniques that don't use SeV vectors, this system does not require multiple cycles of infection. Furthermore, the efficiency of iPSC generation achieved was 20∼100 times higher than that obtained using techniques such as retrovirus, lentivirus, or plasmid vectors [5], [15], [21].

Consideration of the cell source is important when applying iPSC technology to clinical medicine. Although skin fibroblasts are the most common cell type used for generating iPSCs, we consider that peripheral blood cells are preferable because collection is less invasive and is suitable for children and patients with skin or coagulopathy disorders. Here, we demonstrated that the TS12KOS vector generates iPSCs from both skin fibroblasts (∼4%) and peripheral blood cells (∼2%) with high efficiency.

Overexpression of the four ‘reprogramming’ factors needed to generate iPSCs was initially mediated by lentivirus and retrovirus vectors in human skin-derived fibroblasts [5], [15]. Although these gene expression systems are stable, they have two potential problems in that the genes encoding the four factors are integrated into the host genome and remain in the resultant iPSCs, and there is a risk of insertional mutagenesis, which can facilitate tumorigenesis *in vivo* [30]. The development of efficient and safe reprograming methods based on using Cre/loxP recombination systems, adenovirus vector, piggyback transposons, microRNA, and protein has suffered from a low frequency of iPSC colony generation, a need for repetitive induction, and retention of a short length of foreign DNA in the host genome [16]–[18], [31], [32]. A recent study showed that episomal plasmid vectors, which rarely integrate into the host genome, can be used to generate iPSCs from blood cells; however, the efficiency was low (∼0.1%) and factors such as p53 knock-down and the transient expression of Epstein-Barr virus nuclear antigen (EBNA) were required in addition to the four reprogramming factors [21]. To overcome these remaining iPSC issues, in this study we developed a new type of SeV vector that can easily and efficiently provide transgene-free iPSC lines from human and chimpanzee blood cells. SeV vectors are minus-strand RNA viruses that express a gene of interest without integration into the host genome [33]. Thus, our vector can overcome the obstacles described above.

The iPSCs derived from nonhuman primates are useful tools for regenerative medicine research because of the similarities in anatomy and physiology between those mammalian species and human, and chimpanzee is a particularly useful such model [9], [10]. Chimpanzee and human share much of their genomic DNA sequences, with only ∼1.2% difference [34]. To this end, we also generated transgene-free iPSC lines from chimpanzee blood cells using the new SeV vector. The efficiency of iPSC generation from chimpanzee blood was lower than that from human blood. Stimulation methods of T lymphocytes and human-derived reprogramming factors may affect the efficiency. Nevertheless, further studies are necessary to improve the efficiency of iPSC generation from chimpanzee blood. Recently, other group established the chimpanzee iPSC lines from skin-derived fibroblasts with the retrovirus vector [35]. However, it is difficult in collecting many chimpanzee-derived fibroblasts because of breeding limitation for medical use. Our new vector can easily provide the transgene-free iPSCs from the chimpanzee blood cells that is less limited than other tissues.

The chimpanzee iPSC lines established here showed the requisite pluripotency and other features of established iPSCs and thus could provide us with alternative and highly valuable tools. First, they could be used to generate fresh chimpanzee cells, which are valuable cell models and difficult to derive from the animal itself due to breeding limitations. For example, neural cells derived from chimpanzee iPSCs could permit us to study neural development and function, and thus facilitate discovery and increased understanding of the substantial neurological differences between human and chimpanzee despite the largely identical genomes.

Furthermore, the technology used herein could be applied to generate iPSCs from nonhuman primates other than chimpanzees. SeV can infect the blood cells of rhesus monkeys, Macaca fascicularis and marmosets, suggesting that our new vector could also easily induce cell reprogramming and iPSC generation from their blood cells [36], [37]. These approaches are expected to improve our ability to better understand and interrogate the distinguishing traits of human and potentially open up a new field in studying the development of human capacity during evolution.

## Methods

### iPSC Generation and maintenance

All experimental procedures of human samples were approved by the ethics committees, “ Ethics committee for Epidemiological and General Research at the Faculty of Life Science, Kumamoto University”, “Ethics committee for Human genome and Gene analysis Research at the Faculty of Life Sciences, Kumamoto University” and “Ethics committee for clinical research and advanced medical technology, Kumamoto University” (approval numbers 318, 153 and 1018, respectively) and conformed to the human sample use guidelines of the ethics committees. After explaining our study, the volunteers agreed with our study and signed the sheets of written informed consent.

Human skin biopsies and peripheral blood were collected from healthy volunteers following informed consent under protocols approved by the ethics committee assigning authors. For human fibroblast generation, skin samples were minced and cultured in Dulbecco's modified essential medium (DMEM, Life Technologies) supplemented with 10% fetal bovine serum (FBS). The subsequent fibroblast cultures were expanded for iPS cell induction.

The use of the chimpanzees during the experimental period adhered to the Guidelines for Care and Use of Nonhuman Primates (2010) of the Primate Research Institute of Kyoto University. The ethical committee of the Primate Research Institute of Kyoto University approved the protocols of experimental procedures in this study (Permit Number: 2012-134). Blood samples were obtained from two individuals, Puchi (GAIN-ID:0436) and Ai (GAIN-ID:0434), for routine veterinary and microbiological examination under ketamine anesthesia, and all efforts were made to minimize suffering.

To generate iPS cells from peripheral blood cells, mononuclear cells (MNCs) were isolated by Ficoll gradient method. To stimulate T lymphocytes, MNCs were cultured on anti-CD3 antibody-coated dishes in KBM502 medium (KOHJIN BIO) or RPMI-1640 medium (Invitrogen) supplemented with 10% FBS and IL-2 for five days. In some experiments, instead of anti-CD3 antibody (eBioScience), 10 µg/ml Phytohemagglutinin (PHA, SIGMA) or 1 µg/ml Concanavalin A (Con A, SIGMA) were used for the stimulation.

iPSCs were generated from human skin-derived fibroblasts and human- and chimpanzee-stimulated T lymphocytes as described previously [20]. Briefly, 1×10^5^ of the MNCs per well of 48-well plate and 5×10^5^ cells of the fibroblasts per well of 6-well plate were seeded one day before infection and then were infected with Sendai virus (SeV) vectors at various multiplicity of infection (MOI) including three, ten and thirty. After two-day culturing for blood cells and seven-day culturing for fibroblasts, the cells infected were harvested by trypsin and re-plated at 5×10^4^ cells per 60 mm dish on the mitomycin C (MMC)-treated mouse embryonic fibroblast (MEF) feeder cells. Next day, the medium was replaced in human iPS cell medium. The cultures with new Sendai virus infection were incubated at 36°C for one week. From 18 to 25 days after infection, colonies were picked up and re-cultured again in the iPS cell medium. In some experiments, FGF2 concentration was modified from 5 ng/ml to 30 ng/ml and NaB was added on day 2 in the iPSC induction. To remove Sendai virus, the temperature of culture shifts from 37°C to 38°C for three days at passage 1 or 2 of iPSCs.

Human and chimpanzee iPSC lines were maintained on MMC-treated MEF feeder cells in the iPS medium containing DMEM/F12 (SIGMA) supplemented with 20% KNOCKOUT serum replacement (KSR, Invitrogen), 2 mM L-glutamine (Life technologies), 0.1 mM nonessential amino acids (NEAA, SIGMA), 0.1 mM 2-mercaptoethanol (2ME, SIGMA), 0.5% penicillin and streptomycin (Nacalai Tesque, Japan) and 5 ng/ml or 30 ng/ml basic fibroblast growth factor (bFGF, WAKO, Japan).

### Chimpanzee rearing

At the Primate Research Institute of Kyoto University, the subject chimpanzees lived in two mixed-sex groups in an outdoor enclosure that connected to several inside rooms. The outdoor enclosure was separated into two compartments: one was a 700-m^2^ outdoor compound with 15-m-high climbing frames, a small stream and numerous trees; the other was a 250-m^2^ outdoor compound with climbing frames and two small streams. Chimpanzees could freely access the outdoor enclosure and inside room at all times. The chimpanzees were fed seasonal fruits and vegetables, along with monkey pellets three times per day and were provided feeding-enrichment items between meals on a few occasions.

### Generation of Sendai virus (SeV) vectors

Generation and production of temperature-sensitive Sendai virus vectors were performed as described previously [22]. The conventional type of SeV vectors carrying Oct3/4, Sox2, Klf4 and c-Myc were also generated as described previously [22]. To generate TS12 vector, three mutations including D433A, R434A and K437A were introduced into the polymerase-related gene *P*. For TS15 vector generation, three other mutations, Y942H, L1361C, and L1558I, were inserted into polymerase-related genes *L* of TS12. For “three-in-one” vector, human *KLF4, OCT3/4* and *SOX2* genes were inserted between *P* and *M* gene-encoding region in order as described in Fig. 1A. Each gene was sandwiched by *E* (End), *I* (Intervening) and *S* (Start) sequences.

### Karyotype analysis

G band analyses of chromosome were performed by Nihon Gene Research Laboratories. Inc. (Sendai, Japan), according to the manufacturer's protocol.

### DNA and RNA Isolations and PCR

Genomic DNA was extracted from chimpanzee iPSC lines as described previously [38]. Total RNA was purified with Sepasol Super G reagent (Nacalai Tesque, Japan). Total RNA was transcribed to DNA with Superscript III (Invitrogen) and randam primers (Invitrogen). Genomic PCR and RT-PCR was performed with QuickTaq (TOYOBO, Japan) as described previously [24], [38]. Primers used for Oct3/4, Sox2, Klf4 and c-Myc were designed to detect the expressions of endogenous genes, but not of transgenes. To detect SeV genome, nested RT-PCR was performed. The sequences of primers and amplification conditions are listed in **Table S1**.

### Cell staining and Immunocytochemistry

Alkaline phosphatase staining was performed using the Leukocyte Alkaline Phosphatase kit (SIGMA). For immunocytochemistry, cells were fixed with PBS containing 4% paraformaldehyde for 30 min at 4°C. For the molecules localized in nucleus, samples were treated with 0.2% Triton X-100 for 15 min at room temperature (RT). The cells were washed three times with PBS containing 2% FBS and then incubated overnight at 4°C in PBS containing 2% FBS with primary antibodies. The list of the primary and secondary antibodies is described in **Table S2**.

### Southern blotting

TCR probe was amplified by PCR as described previously using cDNA of human peripheral blood mononuclear cells and labeled with α^32^P-dCTP by BcaBEST labeling Kit (Takara Bio Co. Ltd). Commercially available membranes–Hybond-N^+^ (GE Healthcare) were used and hybridization was performed as described previously [38].

### Differentiation into three germ layer cells

Mesoderm-like cell cultures were specified based upon a previously described protocol [39]. For the embryoid body (EB) formation, iPSC clusters were transferred to low attachment dishes in DMEM/F12 (SIGMA) supplemented with 20% KSR (Invitrogen) and 10 ng/ml BMP4 (R & D). Next day, the formed EBs were transferred to collagen IV-coated tissue culture plates (BD) in the medoserm induction medium containing alpha-MEM supplemented with 10% FBS, 0.1 mM 2ME, 3 ng/ml Activin (R & D) 10 ng/ml BMP4 and 5 ng/ml bFGF (WAKO). On day 4, the cells were harvested and analyzed for BRACHYURY expression. For endoderm-like cell induction, the culture medium of semi-confluent human iPS cells were switched from the iPS medium to the definitive endoderm differentiation medium containing RPMI1640 supplemented with 2% B27 (Life technologies), 100 ng/ml Activin A (R & D) and 1 mM Sodium butyrate (NaB, SIGMA). The NaB concentration is changed in 0.5 mM on day 2. On day4, the cells were stained with anti-SOX17 antibody.

For neural cell induction, the iPSC clusters were plated onto Geltrex plates (Life Technologies). 24 hours later, the culture medium was switched from the iPSC medium to PSC Neural Induction Medium (Life Technologies) containing Neurobasal medium and PSC neural induction supplement [40]. On day 7, the cells were dissociated with TrypLE express (Life Technologies) and re-seeded onto Geltrex-coated plates in NSC expansion medium containing 50% Neurobasal medium, 50% Advanced DMEM/F12, neural induction supplement and 5 µM Rock inhibitor, Y-27632. On day 14, the cells were stained with anti-betaIII tubulin antibody.

### Teratoma formation

Human and chimpanzee iPSC lines grown on MEF feeder layers were collected by collagenase IV treatment and injected into the testis of NOD-SCID immunodeficient mice. Palpable tumors were observed about 12–16 weeks after injection. Tumor samples were collected, fixed in 10% formalin, and processed for paraffin-embedding and hematoxylin-eosin staining following standard procedures.

### Microarray analysis

Two hundred fifty ng of total RNA from the chimpanzee iPSCs were labeled with biotin and fragmented according to the manufacturer's protocol (3′ IVT Express kit, Affymetrix). Then, samples were hybridized to a GeneChip Human Genome U133 Plus 2.0 (Affymetrix) Arrays were scanned with a GeneChip Scanner 3000(Affymetrix). Data were analyzed using GeneSpring GX 12.5 software (Agilent technologies). Each chip is normalized to the median of the measurements.

## Supporting Information

Figure S1 iPS cell generation with SeV vector carrying *L-Myc* and *Glis1*. (**a**) Schematic structure of Sendai virus (SeV) vectors carrying *L-My*c and *Glis1*. The exogenous *L-Myc* cDNA is inserted between HN and L positions in TS15 vector. *Glis1* cDNA were inserted between HN and L positions in conventional and TS15 SeV vectors, HNL/TS Glis1 and HNL/TS15 Glis1. We also generated other two vectors, +18/TS Glis1 and +18/TS15 Glis1, which carry Glis1 in the downstreams of Leader in conventional and TS15 SeV vectors. (**b**) Efficiency of iPS cell generation with Myc vectors. The efficiency of iPS cell generation is much lower by *L-Myc* SeV vector than by *c-Myc* SeV vector. iPS cell colonies were identified on day 28 of induction by the appearance of alkaline phosphatase-positive (AP+) colonies with ES cell-like colony morphology. Colony number (right picture) were counted and summarized in left graph. MOI: multiplicity of infection. (**c**) Efficiency of iPS cell generation with various Glis1 vectors. iPS cells were generated with the three factors (K, O, S) plus *Glis1* in the presence (left graph) and absence (right graph) of *c-Myc*. Both cases showed that *Glis1* in SeV vectors did not enhance the efficiency of iPS cell generation.
(TIF)

Figure S2 Experimental design of iPSC induction from chimpanzee blood cells. After collecting mononuclear cells (MNCs) from the chimpanzee blood, MNCs were stimulated with anti-CD3 antibody (Exp. 1) or Con A (Exp. 2 and 3) for five days. One day later after the infection of the sendai virus carrying *OCT3/4*, *KLF4*, *SOX2* and *cMYC*, the cells were transferred on the MEFs with 5 ng/ml (Exp. 1) or 30 ng/ml (Exp.2 and 3) FGF2.
(TIF)

Figure S3 Comparing the chimpanzee with human conditions in iPSC generation. Using human blood cells from two volunteers (volunteer 4 and 5), the condition of chimpanzee with 30 ng/ml FGF2 is compared with that of human with 5 ng/ml in iPSC generation. The efficiency of iPSC generation with 30 ng/ml FGF is slightly but not significantly lower than that with 5 ng/ml FGF2.
(TIF)

Table S1 Sequences and PCR conditions of primers sets for PCR.
(DOCX)

Table S2 List of antibodies and their conditions for staining.
((DOCX))
