# Supplementary figures and images for: New Type of Sendai Virus Vector Provides Transgene-Free iPS Cells Derived from Chimpanzee Blood
Source: PLoS One. 2014 Dec 5;9(12):e113052. doi: 10.1371/journal.pone.0113052 (PMC4257541; doi:10.1371/journal.pone.0113052)

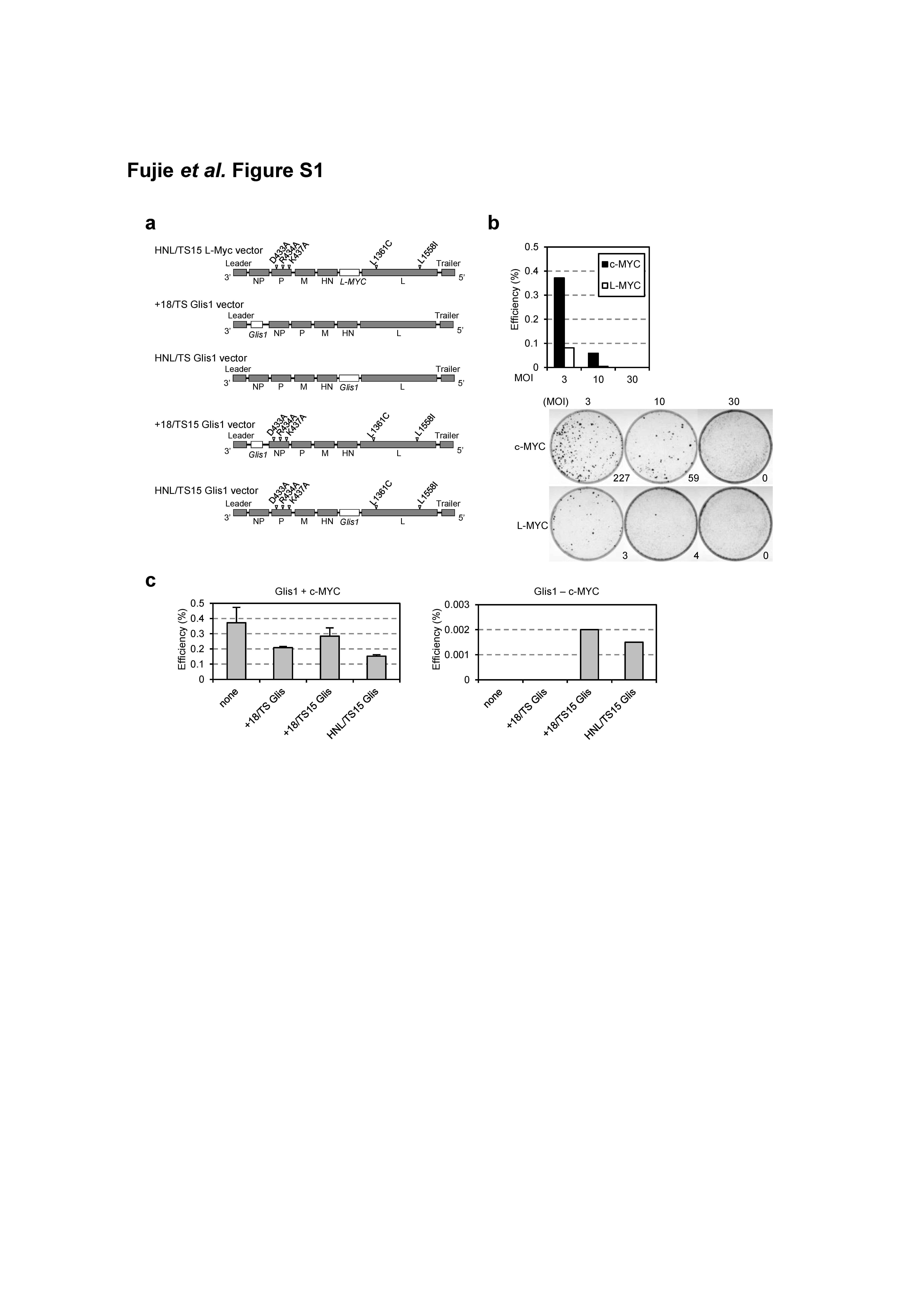

Supplement: Figure S1 — iPS cell generation with SeV vector carrying L-Myc and Glis1. (a) Schematic structure of Sendai virus (SeV) vectors carrying L-Myc and Glis1. The exogenous L-Myc cDNA is inserted between HN and L positions in TS15 vector. Glis1 cDNA were inserted between HN and L positions in conventional and TS15 SeV vectors, HNL/TS Glis1 and HNL/TS15 Glis1. We also generated other two vectors, +18/TS Glis1 and +18/TS15 Glis1, which carry Glis1 in the downstreams of Leader in conventional and TS15 SeV vectors. (b) Efficiency of iPS cell generation with Myc vectors. The efficiency of iPS cell generation is much lower by L-Myc SeV vector than by c-Myc SeV vector. iPS cell colonies were identified on day 28 of induction by the appearance of alkaline phosphatase-positive (AP+) colonies with ES cell-like colony morphology. Colony number (right picture) were counted and summarized in left graph. MOI: multiplicity of infection. (c) Efficiency of iPS cell generation with various Glis1 vectors. iPS cells were generated with the three factors (K, O, S) plus Glis1 in the presence (left graph) and absence (right graph) of c-Myc. Both cases showed that Glis1 in SeV vectors did not enhance the efficiency of iPS cell generation. (TIF) [file pone.0113052.s001.tif]

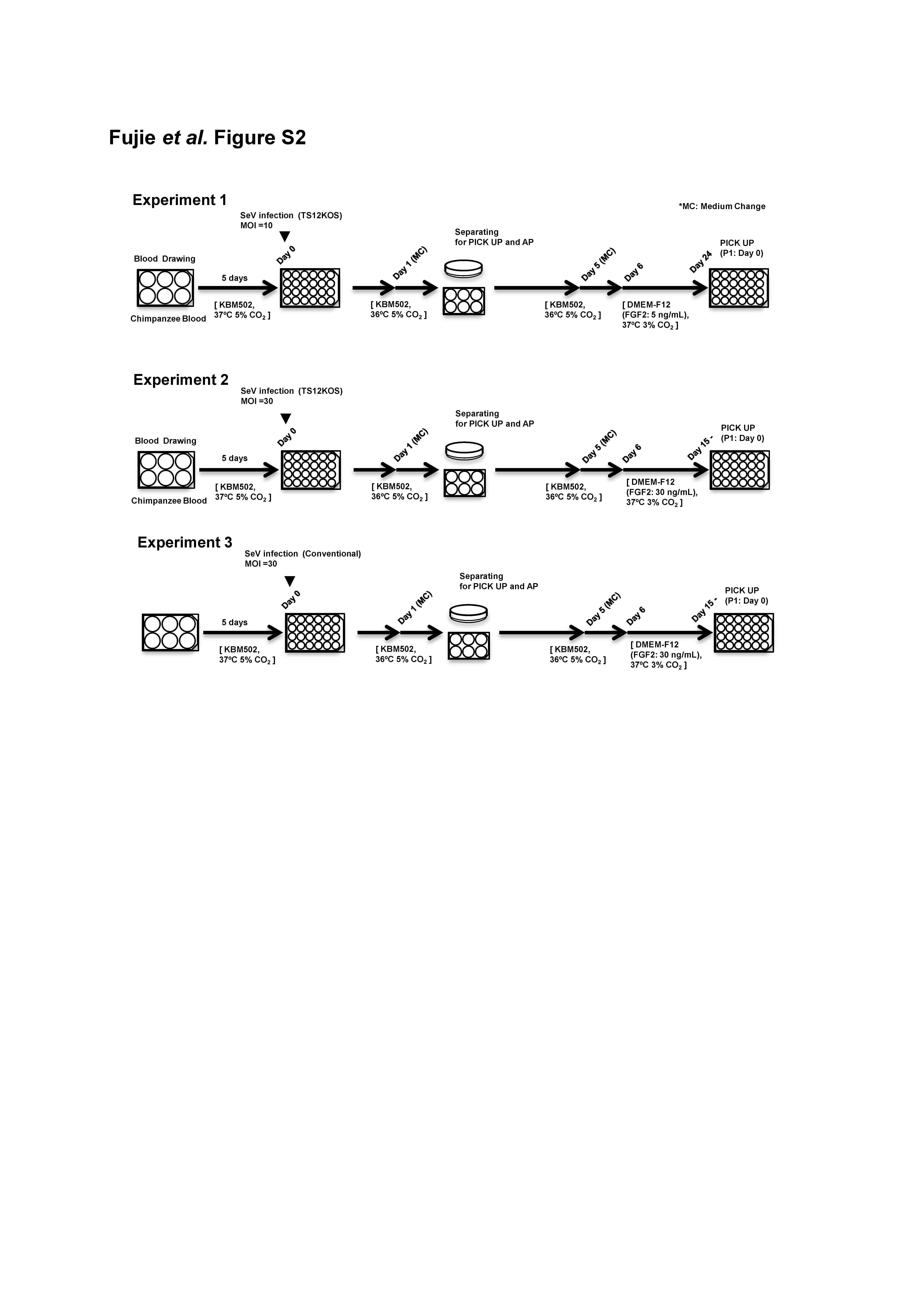

Supplement: Figure S2 — Experimental design of iPSC induction from chimpanzee blood cells. After collecting mononuclear cells (MNCs) from the chimpanzee blood, MNCs were stimulated with anti-CD3 antibody (Exp. 1) or Con A (Exp. 2 and 3) for five days. One day later after the infection of the sendai virus carrying OCT3/4, KLF4, SOX2 and cMYC, the cells were transferred on the MEFs with 5 ng/ml (Exp. 1) or 30 ng/ml (Exp.2 and 3) FGF2. (TIF) [file pone.0113052.s002.tif]

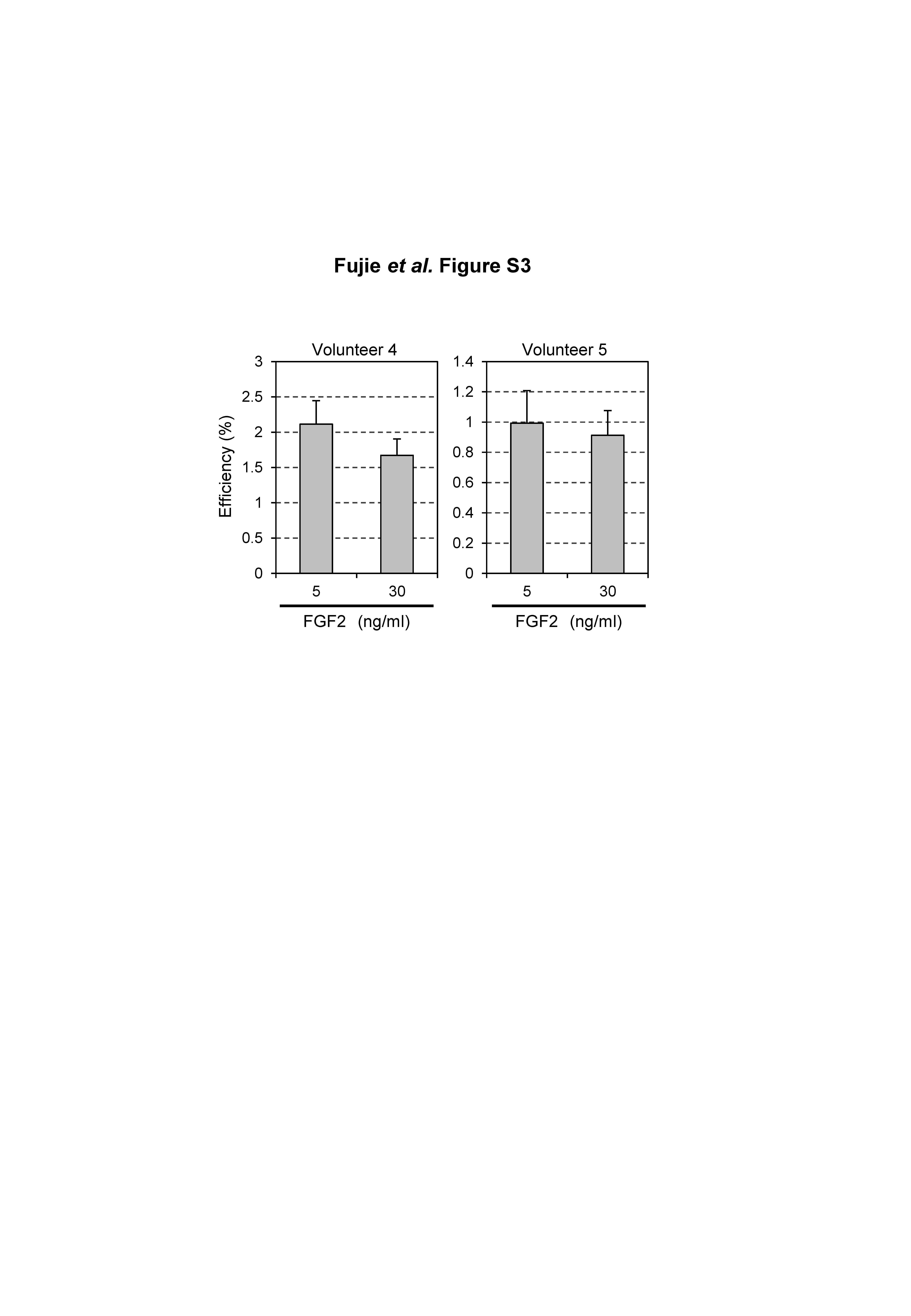

Supplement: Figure S3 — Comparing the chimpanzee with human conditions in iPSC generation. Using human blood cells from two volunteers (volunteer 4 and 5), the condition of chimpanzee with 30 ng/ml FGF2 is compared with that of human with 5 ng/ml in iPSC generation. The efficiency of iPSC generation with 30 ng/ml FGF is slightly but not significantly lower than that with 5 ng/ml FGF2. (TIF) [file pone.0113052.s003.tif]
