# Supplementary material for: New Type of Sendai Virus Vector Provides Transgene-Free iPS Cells Derived from Chimpanzee Blood
Source: PLoS One. 2014 Dec 5;9(12):e113052. doi: 10.1371/journal.pone.0113052 (PMC4257541; doi:10.1371/journal.pone.0113052)
Supplement: Table S1 — Sequences and PCR conditions of primers sets for PCR. (DOCX) [file pone.0113052.s004.docx]

**Fujie et al.　Table S1**

The sequences of primer sets

| Genes | Sequences  (Forward; F, Reverse; R) | Annealing (℃) | Cycle | Product  size (bp) |
| --- | --- | --- | --- | --- |
| SeV | F: GGATCACTAGGTGATATCGAGC  R: ACCAGACAAGAGTTTAAGAGATATGTATC | 58 | 30 | 181 |
| Nested | F: TCGAGCCATATGACAGCTCG  R: GAGATATGTATCCTTTTAAATTTTCTTGTCTTCTTG | 58 | 30 | 148 |
| OCT3/4 | F: GACAGGGGGAGGGGAGGAGCTAGG  R: CTTCCCTCCAACCAGTTGCCCCAAAC | 55 | 33 | 144 |
| SOX2 | F: GGGAAATGGGAGGGGTGCAAAAGAGG  R: TTGCGTGAGTGTGGATGGGATTGGTG | 55 | 33 | 151 |
| KLF4 | F: GATTACGCGGGCTGCGGCAAAACCTACACA  R: TGATTGTAGTGCTTTCTGGCTGGGCTCC | 56 | 35 | 357 |
| c-MYC | F: GCGTCCTGGGAAGGGAGATCCGGAGC  R: TTGAGGGGCATCGTCGCGGGAGGCTG | 56 | 33 | 328 |
| NANOG | F: CAGCCCCGATTCTTCCACCAGTCCC  R: CGGAAGATTCCCAGTCGGGTTCACC | 60 | 30 | 391 |
| GDF3 | F: CTTATGCTACGTAAAGGAGCTGGG  R: GTGCCAACCCAGGTCCCGGAAGTT | 56 | 35 | 631 |
| REX1 | F: CAGATCCTAAACAGCTCGCAGAAT  R: GCGTACGCAAATTAAAGTCCAGA | 55 | 30 | 306 |
| SALL4 | F: AAACCCCAGCACATCAACTC  R: GTCATTCCCTGGGTGGTTC | 58 | 30 | 138 |
| DNMT3b | F: TGCTGCTCACAGGGCCCGATACTTC  R: TCCTTTCGAGCTCAGTGCACCACAAAAC | 55 | 33 | 242 |
| β-ACTIN | F: CAACCGCGAGAAGATGAC  R: AGGAAGGCTGGAAGAGTG | 60 | 25 | 455 |
| Chimp Chr 2a | F: ATTGGCCATCTCTTCATGCCCTGAG  R: ACTTGCTAATGCATTCCCTGATGGG | 57 | 30 | Chimp 782  Human 203 |
| Chimp Chr 11 | F: TGATTCAAATGACCTTCGTGGGTGC  R: ACCAGGAGTGGGATCTACTTTCTGG | 57 | 30 | Chimp 472  Human 245 |
| Chimp Chr 12 | F: TCACAAGAACTACAGTCCTCATCTC  R: GGTATGTATCTGAACCTGATTAGGC | 57 | 30 | Chimp 504  Human 278 |
| Cβ-1 | F: CTTCGAATTCCAGAGGACCTGAACAAGGTG  R: CCACAAGCTTGCTCTACCCCAGGCCTCGGC | 55 | 40 | 398 |
| Jβ-1-1 | F: GGGGGCTCCAAGCTTGACTCGGG  R: AGATGAATTCATGAGCAGAGTTCTGTGCTGG | 55 | 40 | 435 |
| Jβ-1-2 | F: CCATAAGCTTAAGCCTCAGAATCTATGCTAG  R: CATAGAATTCAGGAATGAAAAGGATTGTCACCAC | 55 | 40 | 435 |
