# Supplementary material for: New Type of Sendai Virus Vector Provides Transgene-Free iPS Cells Derived from Chimpanzee Blood
Source: PLoS One. 2014 Dec 5;9(12):e113052. doi: 10.1371/journal.pone.0113052 (PMC4257541; doi:10.1371/journal.pone.0113052)
Supplement: Table S2 — List of antibodies and their conditions for staining. ((DOCX)) [file pone.0113052.s005.docx]

**Fujie et al.　Table S2**

List for antibodies applied.

| Antibody | Species | Dilution | Vendor |
| --- | --- | --- | --- |
| Anti-Nanog | Goat | 1:1000 | R&D systems |
| Anti-Oct3/4 | Mouse | 1:500 | Santa Cruz |
| Anti-SSEA4 | Mouse | 1:500 | MILLIPORE |
| Anti-TRA-1-60 | Mouse | 1:500 | MILLIPORE |
| Anti-Sox17 | Mouse | 1:500 | R&D systems |
| Anti-Brachyury | Mouse | 1:500 | R&D systems |
| Anti-β-III-tubulin | Mouse | 1:500 | MILLIPORE |
| Alexa 488-conjugated anti-mouse IgG | goat | 1:1000 | Invitrogen |
| Alexa 488-conjugated anti-goat IgG | donkey | 1:1000 | Invitrogen |
